# Changes in Obesity Prevalence Attributable to Ultra-Processed Food Consumption in Brazil Between 2002 and 2009

**DOI:** 10.3389/ijph.2022.1604103

**Published:** 2022-05-20

**Authors:** Maria Laura Louzada, Eurídice Martinez Steele, Leandro F. M. Rezende, Renata Bertazzi Levy, Carlos Augusto Monteiro

**Affiliations:** ^1^ School of Public Health, University of São Paulo, São Paulo, Brazil; ^2^ Center for Epidemiological Research in Nutrition and Health, School of Public Health, University of São Paulo, São Paulo, Brazil; ^3^ Departamento de Medicina Preventiva, Escola Paulista de Medicina, Universidade Federal de São Paulo, São Paulo, Brazil; ^4^ Faculty of Medicine, University of São Paulo, São Paulo, Brazil

**Keywords:** obesity, Brazil, ultra-processed foods, population attributable fraction, epidemiology, nutrition surveys

## Abstract

**Objectives:** To quantify the impact of temporal changes in the consumption of ultra-processed foods on obesity trends in Brazil between 2002 and 2009.

**Methods:** We analyzed data from two Household Budget Surveys carried out in 2002/2003 (*n* = 182,333) and 2008/2009 (*n* = 190,159), which provided information on household food acquisition and individuals’ weight and height. We examined the association between ultra-processed foods consumption and obesity and quantified the fraction of increase in obesity prevalence attributable to the rise in the consumption of ultra-processed foods.

**Results:** From 2002 to 2009, there was an increase in the obesity prevalence from 9.9% (95% CI 9.3; 10.4) to 13.2% (12.8; 13.7) while the contribution of ultra-processed foods to total energy consumption raised from 14.3% (13.4; 15.1) to 17.3% (16.5; 18.1). Ultra-processed foods consumption was positively associated with obesity prevalence. More than one quarter (28.6%) of the increase in obesity prevalence was attributable to the rise in the consumption of ultra-processed foods in the period.

**Conclusion:** We found that the rise in the consumption of ultra-processed foods played a major role on the increase of obesity epidemic in Brazil.

## Introduction

Obesity epidemic is currently a public health challenge worldwide. Since 1980, its prevalence has doubled in more than 70 countries and has steadily increased in most other countries. In 2017, overweight (body mass index—BMI ≥25 kg/m^2^) was the fourth most relevant risk factor for the global burden of disease, accounting for more than 4 million deaths and almost 150 million disability-adjusted life years (DALYs) (1).

In Brazil, nationally representative surveys have shown that obesity prevalence in all income classes and age groups has been continuously and significantly increasing in last decades (2, 3). Between 1974 and 2009, the obesity prevalence in children aged 5–9 years increased from 2.9% to 16.5% among boys and from 1.8% to 11.8% among girls. For adolescents (aged 10–19 years), the prevalence varied from 0.4% to 5.9% in males and from 0.7% to 4.0% in females in the same period (2). In adults aged 20 years or older, the obesity prevalence increased by more than eightfold (2.8%–22.8%) among men and threefold (8.0%–30.2%) among women from 1974 to 2019 (3).

The increasing obesity prevalence in Brazil and worldwide has occurred in parallel with dramatic transformations in the globalizing food system. These changes are mainly characterized by the gradual weakening of traditional food patterns, based on fresh or minimally processed foods, with the concomitant increase in the consumption of ultra-processed foods (4, 5). Ultra-processed foods are industrial formulations typically ready for consumption made of numerous ingredients, often obtained from high-yield crops, such as sugars and syrups, refined starches, oils and fats, protein isolates, in addition to remains of intensive animal farming. These formulations are made to be visually attractive, have a seductive aroma, and very intense or even “irresistible” flavors, using sophisticated combinations of flavorings, dyes, emulsifiers, sweeteners, thickeners, and other additives that modify the sensory attributes. Natural or minimally processed foods represent a reduced proportion or are not even present in the ultra-processed ingredients list. Examples are cookies, candies, salty snacks, soft drinks, artificial juices, and several ready-to-eat meals (6). Food sales statistics suggest that sales of ultra-processed foods have been expanding intensively in many countries around the world since the 1990s, with particular intensity in middle-income countries (4). In Brazil, household food acquisition surveys showed that the dietary share of ultra-processed foods increased from 14.3% in 2002/2003 to 19.4%, in 2017/2018 (7) and that it was cross-sectionally associated with the occurrence of obesity (8).

Increasing epidemiological evidence shows an association between the consumption of ultra-processed foods and increased risk of obesity (9, 10). Ultra-processed foods are convenient and palatable and replace meals based on fresh or minimally processed foods. These foods have higher energy density, more free sugar and unhealthy fats, and less dietary fiber, protein, micronutrients, and health-protective bioactive compounds than non-ultra-processed foods and their consumption is systematically associated with the deterioration of nutritional dietary quality (11–16). Experimental studies comparing non-ultra-processed foods to ultra-processed foods demonstrated that the latter has low satiety power, induces high glycemic responses (17), is associated with a higher energy intake rate (18), has a higher presence of contaminants newly formed during processing or released from synthetic packaging (19, 20), and may create an intestinal environment that favors microbes promoting inflammatory diseases (21). A recent cross-over randomized trial compared the effect of *ad libitum* diets with more than 80% of energy from ultra-processed foods with *ad libitum* diets without ultra-processed foods. Even with meals designed to offer an equal number of calories and various nutrients, when exposed to the ultra-processed diet, individuals consumed, on average, 508 more calories per day and, as expected, gained on average 1 kg of weight in the period of 2 weeks while, when exposed to the non-ultra-processed diet, they lost 1 kg (22).

Beyond traditional studies of relative risk associated with etiological factors, epidemiological studies can also orient health promotion and public health policies by providing estimates of the population attributable fractions, which answer questions about the relative importance of distinct risk factors on the burden of diseases and conditions (23). Estimating the population attributable fractions has been used to inform managers and decision-makers to assist in public policymaking. For example, the World Health Organization (WHO) provides information on the disease burden attributable to the main modifiable risk factors through the Global Burden of Disease Study*,* and the Pan American Health Organization supports a workgroup studying the estimates of deaths attributable to sodium consumption in the Americas. Despite their relevance, these studies are scarce, particularly in low- and middle-income countries.

The availability of two representative surveys of the Brazilian population, which provide simultaneous information on household food acquisition and the individuals’ nutritional status, allows an unprecedented quantification of the fraction of increase in obesity prevalence attributable to the rise in the consumption of ultra-processed foods in Brazil. Therefore, this study aims to quantify the fraction of increase in obesity prevalence attributable to the rise in the consumption of ultra-processed food in Brazil between 2002/2003 and 2008/2009.

## Methods

### Data Source and Sampling

We analyzed data from two Household Budget Surveys (POF) conducted in Brazil in 2002/2003 and 2008/2009, which are the most up-to-date national surveys to simultaneously provide information on household food acquisition and individuals’ weight and height.

Both surveys employed complex sampling plans, with a clustered sampling procedure, based on the random selection of census sectors during the first stage and of households in the second stage. Initially, the census tracts of the country were organized into strata with high geographic and socioeconomic homogeneity. For this, the location of the sectors (region, federation unit, capital or interior, urban, or rural area) and the spectrum of variation of the socioeconomic level of the families were considered. Census tracts in each stratum and households belonging to each census tract were selected. Household interviews within each stratum were evenly distributed over the four year-quarters of the study duration.

The POFs studied probabilistic samples with national representativeness, evaluating 182,333 people from 48,470 households in 2002–2003 and 190,159 people from 55,970 households in 2008–2009. The household strata included in the research sample plan were used as the primary analysis unit for this study. These strata had to be homogeneous in terms of the household geographic location and families’ socioeconomic level, totaling 443 in 2002–2003 and 550 in 2008–2009. The average number of households by each studied stratum was 109.4 (ranging from 9 to 801) in 2002–2003 and 101.7 (ranging from 8 to 796) in 2008–2009.

### Assessment of Ultra-Processed Food Acquisition

In 2002/2003 and 2008/2009, detailed information was recorded on all expenses incurred with the food and beverages purchase for consumption at home during seven consecutive days (including the exact purchased quantity for each food item). The same information about non-monetary acquisitions (such as donations and self-production) was also recorded and converted into monetary values. Food consumed by family members outside the home was not recorded in sufficient detail and thus not included in the analysis.

The total quantities purchased of each food item, after excluding the non-edible fraction, were converted to express daily consumption values (i.e., the total amount divided by 7 days). The total daily amount purchased for each food was converted into energy using the Brazilian Table of Food Composition (TBCA) of the University of São Paulo (USP), Food Research Center (FoRC), Version 7.0. São Paulo, 2019 [available at: http://www.fcf.usp.br/tbca].

The consumption items were subsequently divided into four groups based on the NOVA food classification system (6), which takes into account the extent and purpose of industrial food processing: Group 1—Natural or minimally processed foods; Group 2—Processed culinary ingredients; Group 3—Processed foods; Group 4—Ultra-processed foods.

Then, the total calories acquired by all households were distributed according to the four food groups and the percentage of contribution of ultra-processed foods to the total purchased energy was calculated.

### Obesity Assessment

In the 2002/2003 and 2008/2009 POFs, measurements of weight and height of all residents of the household were obtained and recorded in the questionnaires filled by the research agents, following standardized measurement techniques (24–26).

Weight was measured in kilograms (kg) with portable electronic scales with a maximum capacity of 150 kg and 100 g (g) intervals. Height was expressed in centimeters (cm), using length (lying down) as a measure in children aged zero to 23 months and height (standing) in individuals aged 24 months or more. To measure the length, children’s anthropometers were used with a capacity of up to 105 cm and a scale in millimeters, while the height was measured using portable stadiometers with a 200 cm retractable tape with 0.1 cm precision. After data collection, imputation procedures were applied to deal with non-answers or answers associated with rejected values in a critical review phase.

Based on these measures, the BMI of all individuals was calculated by dividing their weight (in kg) by their squared height (in meters). The populational nutritional status of children and adolescents was based on the BMI-for-age indicator in line with the WHO (27, 28). Obesity was ascertained by BMI-for-age equal to or greater than 2 z scores (28). In the adult population aged 20 years or more, obesity was considered BMI equal to or greater than 30 kg/m^2^ (29). Then, the prevalence of obesity in each stratum was calculated (primary unit of analysis of the study).

### Covariates

Data on household income, expenses, and other information on the household characterizations (household setting and region of the country) and their residents (sex, age) were collected by trained interviewers using standardized questionnaires.

### Data Analysis

First, the temporal variation in both obesity prevalence and the consumption of ultra-processed foods (% of total energy) was assessed by comparing mean estimates (and their corresponding 95% confidence intervals) obtained from the 2002/3 and the 2008/9 surveys. The statistical significance of the differences between the two estimates was assessed by the test of means for independent samples (Student’s t-test).

Subsequently, we examined, in the two surveys, the cross-sectional association between the consumption of ultra-processed foods (% of total energy) and the prevalence of obesity. This analysis was carried out using a multiple linear regression model for each survey. These models, hereinafter referred to as the 2002/3 model and the 2008/9 model, generated adjusted coefficients, for each survey, that represents, in cross sectional associations, the prevalence increase of obesity for each percentage point increase in consumption of ultra-processed foods (% of total energy). In these analyses, we considered as potential confounding factors sociodemographic variables frequently associated with food consumption and nutritional status, such as country’s region (North, Northeast, South, Southeast, and Midwest), household setting (urban/rural), household income per capita, sex, and age, the last two expressed as a proportion of women, the elderly, and children in the stratum. In addition, the mean percentage of expenditures on food outside the home was also included as a covariate.

Finally, we quantified the fraction of the increase in obesity prevalence attributable to the rise in the consumption of ultra-processed foods (% of total energy) in the period using the methods described by Monteiro et al. (30). Briefly, we calculated the difference between the predicted values for the mean prevalence of obesity when the 2008/9 model was applied successively to the POF database 2008/9 itself and to the POF database 2002/3 and, similarly, the difference between the predicted values for the mean prevalence of obesity when the 2002/3 model was applied successively to the POF database 2002/3 and to the POF database 2008/2009.

In other words, we first ran the 2008/2009 linear regression model and estimated the multiple-adjusted predicted values for the mean prevalence of obesity in 2008/2009. Next, we applied the equation obtained in the 2008/2009 multiple-adjusted regression model to the 2002/2003 ultra-processed food consumption values and estimated the multiple-adjusted predicted values for the mean prevalence of obesity in 2002/2003. Thus, we “fixed” the equation obtained in the regression model and calculated the difference between the multiple-adjusted predicted values of the prevalence of obesity based only on the variation of the magnitude of consumption of ultra-processed foods between the years of 2002/2003 and 2008/2009. Considering that the magnitude of the association between the consumption of ultra-processed foods and the prevalence of obesity (slopes) was not the same in 2002/2003 and 2008/2008, the process was repeated with the 2002/2003 association model applied to the ultra-processed foods consumption values of 2008/2009. The average of these differences was considered the fraction of increase in the prevalence of obesity attributable to the increase in the consumption of ultra-processed foods (% of the total energy).

The obesity prevalence in the counterfactual scenario in which the distribution of consumption of ultra-processed foods in 2008/9 had remained the same as in 2002/3 was then calculated by simply subtracting the fraction of increase in the prevalence of obesity attributable to the increase in the consumption of ultra-processed foods (% of the total energy) from the observed obesity prevalence in 2008/9. Then, we calculated the proportion (%) of the increase in the prevalence of obesity attributable to the increase in the consumption of ultra-processed foods (% of the total energy) as:

$$\frac{\text{FAP∗}100}{\text{O}}$$

In which FAP = fraction of increase in the prevalence of obesity attributable to the increase in the consumption of ultra-processed foods (% of the total energy).

O = observed increase in the prevalence of obesity in the period

Sensitivity analyses considered only participants older than 5 years of age, as measures of weight and height and the obesity ascertained are less precise in younger than 5. All analyses took into account the weighting factors of each survey as well as the effect of the complex sampling strategy on the standard error of estimates. All statistical analyses in the present study were performed using Stata software, version 15.

## Results

Table 1 presents the estimates for the consumption of ultra-processed foods (% of total purchased energy) and the prevalence of obesity in the strata of Brazilian households for 2002/3 and 2008/9. From 2002 to 2009, there was an increase in the prevalence of obesity from 9.91% (95% CI 9.38; 10.44) to 13.29% (12.84; 13.74) while the contribution of ultra-processed foods to total energy consumption increased from 14.30% (13.45; 15.14) to 17.31% (16.52; 18.14) (see also a figure of these trends in the Supplementary Material).

**TABLE 1:** Consumption of ultra-processed foods (% of total energy) and prevalence of obesity. Brazilian households strata in 2002/3 (*n* = 443) and 2008/9 (*n* = 550)^a^ (Brazil 2002/3 and 2008/9).

Variable	2002/3		2008/9	
	Mean	95% CI	Mean	95% CI
Prevalence of obesity (%)	9.91	(9.38; 10.44)	13.29	(12.84; 13.74)^b^
Consumption of ultra-processed foods (% of total energy)	14.30	(13.45; 15.14)	17.31	(16.52; 18.14)^b^

a The average number of households by each stratum was 109.4 in 2002–2003 and 101.7 in 2008–2009.

b Statistically significant difference between the two periods (p < 0.005).

Table 2 describes the association between consumption of ultra-processed foods and obesity prevalence in 2002/3 and 2008/9. In both surveys, the consumption of ultra-processed foods was directly and significantly associated with the prevalence of obesity. A 1% increase in the consumption of ultra-processed foods (% of total energy) was associated with the increase of 0.71 pp (95% CI 0.49; 0.92) in the obesity prevalence in 2002/3 and 0.88 pp (95% CI 0.61; 1.13) in 2008/9.

**TABLE 2:** Multiple-adjusted^a^ regression coefficients between the consumption of ultra-processed foods (% of total energy and the prevalence of obesity (%). Brazilian households strata in 2002/3 (*n* = 443) and 2008/9 (*n* = 550) (Brazil 2002/3 and 2008/9).

Survey year	Coefficients^b^	95% CI	*p* Value
2002/3	0.71	0.49; 0.92	<0.001
2008/9	0.88	0.61; 1.13	<0.001

a Obtained with linear regression models adjusted for household income per capita, setting (urban/rural), region of the country, percentage of expenditure on eating out of home, proportion of children, women and the elderly in stratum.

b The coefficients represents the increase in the prevalence of obesity (%) for each percentage point increase in consumption of ultra-processed foods (% of total energy).

Table 3 compares the predicted values for the mean prevalence of obesity when the adjusted multiple regression models of each survey are applied, successively, to the database of the survey that generated the model and to the database of the other survey. When considering the 2002/3 model, the predicted prevalence of obesity was 10.15% (95% CI 9.79; 10.53) using the 2002/3 ultra-processed food consumption distribution and 10.53% (95% CI 10.38; 10.69) using the 2008/9 ultra-processed food consumption distribution. When considering the 2008/9 model, in turn, the predicted prevalence of obesity was 12.55% (95% CI 12.26; 12.84) using the 2002/3 ultra-processed food consumption distribution and 14.11% (95% CI 13.66; 14.55) using the 2008/9 ultra-processed food consumption distribution.

**TABLE 3:** Prevalence of obesity predicted from two multiple-adjusted linear regression models^a^ according to alternative scenarios regarding the consumption of ultra-processed foods. Brazilian households strata in 2002/3 (*n* = 443) and 2008/9 (*n* = 550) (Brazil 2002/3 and 2008/9).

Survey year	Population distribution of consumption of ultra-processed foods as in	Predicted multiple-adjusted prevalence of obesity (%)		Population distribution of consumption of ultra-processed foods as in	Predicted prevalence of obesity (%)		Difference between predicted values	
		Mean	95% CI		Mean	95% CI	Mean	95% CI
2002/3	2002/3	10.15	9.79; 10.53	2008/9	10.53	10.38; 10.69	0.38	−0.14 to 0.90
2008/9	2002/3	12.55	12.26; 12.84	2008/9	14.11	13.66; 14.55	1.55	0.88 to 2.29

a Models adjusted for household income per capita, setting (urban/rural), region of the country, percentage of expenditure on eating out of home, proportion of children, women and the elderly in stratum.

Thus, it is noteworthy that the exchange of the 2002/3 survey distribution of consumption of ultra-processed foods for the 2008/09 distribution of consumption of ultra-processed foods was associated with an absolute increase in the average prevalence of obesity: 10.53 − 10.15 = 0.38 (ranging from −0.14 to 0.90, 95% CI), when considering the 2002/3 survey association model and 14.11 − 12.55 = 1.55 (ranging from 0.88 to 2.29) when using the 2008/9 association model. The mean of these two increases in obesity prevalence ([0.38 + 1.55]/2), 0.97 (ranging from 0.33 to 1.59), was considered the increase in obesity prevalence attributable to the rise in the consumption of ultra-processed foods in the period.

**FIGURE 1:**
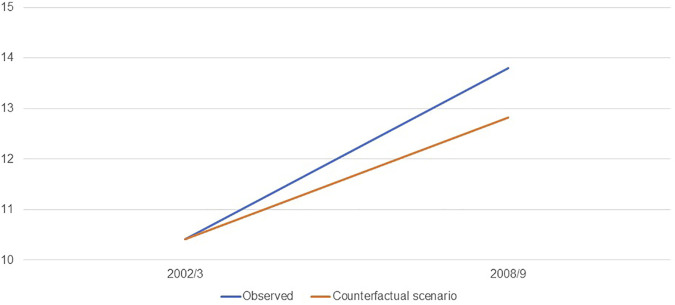
Estimates of the prevalence (%) of obesity in the Brazilian population: observed and counterfactual scenario in which the distribution of consumption of ultra-processed foods in 2008/9 had remained the same as in 2002/3 (Brazil, 2002/3 and 2008/9).

Figure 1 shows the evolution of the prevalence of obesity in Brazil from 2002/3 to 2008/9 in both the observed scenario and the counterfactual scenario in which the distribution of ultra-processed foods consumption in 2008/9 had remained the same as in 2002/3. The prevalence of obesity in 2008/9 in the observed scenario was 13.29% (12.84; 13.74) and in the counterfactual scenario was 12.33% (11.71; 12.97). Therefore, the increase in consumption of ultra-processed foods [calculated as 0.97*100/(13.29 − 9.91)] represented over a quarter (28.65%, ranging from 9.74 to 46.96) of the increase in obesity prevalence in the period. The results were similar when we repeated the analyses excluding participants younger than 5 years old (data not shown).

## Discussion

To the best of our knowledge, this is the first study to quantify the contribution of the increase in the consumption of ultra-processed foods to the rise in the prevalence of obesity in Brazil. From the comparison of two nationally representative surveys, the present study demonstrated that the increase in the consumption of ultra-processed foods in the interval of 7 years (2002–2009) was responsible for more than a fourth of the increase in the prevalence of obesity in the same period.

The rapid increase in the consumption of ultra-processed foods can be explained by changes in the globalized food system. Since the 1980s, neoliberal economic policies and trade agreements designed and promulgated by global organizations supported by important governments have favored the phenomenal expansion of ultra-processed food transnationals. These policies and agreements have deregulated the industry, promoted the capital flow, opened countries to foreign investment, allowed transnational companies to take over national companies, and restricted national governments’ power to introduce statutory policies that limit ultra-processed food consumption. Meanwhile, economic growth and the increase in the average income of some populations made ultra-processed foods accessible to more people. In low- and middle-income countries, such as Brazil, direct and specific advertising targeting lower-income communities also played a role in accelerating the transnationals’ penetration in emerging markets (31).

The increase in the consumption of ultra-processed foods, however, is not restricted to the Brazilian population. For example, household food acquisition data from Canada also denoted that the contribution of ultra-processed foods increased from 24.4% in 1938–1939 to 54.9% in 2001 (32), while Mexico also verified an increase from 10.5% in 1984 to 23.1% in 2016 (33). National food intake surveys indicate that ultra-processed foods already comprise more than half of the total energy consumed in some high-income countries, such as the USA (57%), Canada (51%), and the United Kingdom (56%) (14–16). In addition, ultra-processed foods represent between one-fifth and one-third of the energy consumed in middle-income countries, such as Chile (28%) (12) and Mexico (30%) (13). More recently, analyses of retail food sales databases in 80 countries showed a significant rise in sales of ultra-processed foods between 2002 and 2016, with particular acceleration among middle-income countries. Of note, this increase in sales of ultra-processed foods was positively associated with the temporal increase of the populations’ BMI (34).

The results of this study are even more relevant when considering the impact that obesity may have on individuals’ lives and society in general. Obesity is both a disease and a risk factor for numerous other chronic non-communicable diseases such as cardiovascular disease, cerebrovascular accident, hypertension, dyslipidemia, diabetes, and various types of cancer (35–37). In Brazil, from 1990 to 2015, overweight went from the eighth to the fifth most relevant risk factor for the global disease burden in the country, representing more than 6% of DALYs among men and more than 8% among women (38). Around 45% of diabetes cases in 2008 and 3.8% of the diagnosed cancers in 2012 were attributed to obesity in the country (39, 40). A study using data from Hospital and Outpatient Clinic Information Systems estimated that the cost of treating 26 chronic non-communicable diseases attributable to obesity totaled R$487 million (US$261 million) in 2011, representing 1.9% of the medium and high complexity health care spending in the country (41).

Strategies to reduce or slow the consumption expansion of ultra-processed foods are, therefore, mandatory. However, complex problems have no easy or obvious solutions. Counseling strategies centered on individual responsibility may be successful for some individuals, but they are unlikely to constitute population-wide solutions. Although people have a great responsibility for their food choices, it is essential to recognize that the environment constrains those choices, which may hamper the adoption of a healthy diet. Thus, there is an urge for political actions that prioritize the reduction of obesogenic nature of environments.

Product reformulation, increasingly common in high-income countries is not an effective solution. Changing from one problematic ingredient to another, such as replacing fat by sugar or artificial sweeteners by sugar, neither make ultra-processed foods healthier nor act on other potentially harmful non-nutritional characteristics (42). The successful strategies used to prevent risk factors of chronic non-communicable diseases caused by the excessive use of alcohol and tobacco may also be effective to mitigate the consumption of ultra-processed foods. They include taxation, advertising restrictions, regulation and educational interventions in public and institutional environments, adequate food labeling, and mass educational campaigns. In Brazil, some measures stand out. For instance, the National School Feeding Program guidelines encourage the consumption of fruits and vegetables and other fresh or minimally processed foods, and restricts the purchase of ultra-processed foods (43). Another example is the Dietary Guidelines for the Brazilian Population, whose golden rule is “always prefer natural or minimally processed foods and freshly made dishes and meals to ultra-processed food” (44).

The Lancet Commission on Obesity recently published a report highlighting the conjunction of three pandemics - obesity, malnutrition, and climate change. They defined this simultaneous occurrence as Global Syndemic, considering that they interact with each other in their consequences, and share common drivers, including the underlying characteristics of the food system that drive the increased consumption of ultra-processed foods. The authors propose that several interventions are relevant to the three pandemics, including the establishment of a Framework Convention on Food Systems as the legal framework for healthy, equitable, environmentally sustainable, and economically prosperous food systems (45).

There are some limitations to the interpretation of our findings. In this study, we use data from the POFs that are related to household food purchase instead of individual food consumption. However, previous studies indicate considerable agreement between the estimates obtained by household budget surveys and individual food consumption surveys, particularly concerning the consumption of ultra-processed foods (46, 47). Food purchased and consumed outside the home was not included in the POFs and it is important to emphasize that these foods often belong to the group of ultra-processed products. However, since household consumption is significantly relevant in Brazil (more than 80% of the consumed calories) and the consumption of ultra-processed foods was assessed as a percentage of total energy (and not in absolute values), the estimates presented represent a reliable proxy for total food consumption. In addition, the percentage of expenditures on food outside the home was included as a covariate in the regression models assessing the association between consumption of ultra-processed foods and obesity. Finally, physical activities and smoking status are not usually evaluated in household budget surveys and could not be included as potential confounders in the association between consumption of ultra-processed foods and obesity. However, previous studies conducted in Brazil have shown that patterns of physical activity and smoking are strongly dependent on variables effectively controlled in the analyses, including sex, age, family income, setting, and region of the country (48, 49). However, we cannot exclude the possibility of residual confounding due to physical activity and smoking.

Likewise, our study has significant strengths. The probabilistic nature of the two surveys, their comparability regarding the procedures for collecting and analyzing anthropometric data and food acquisition, and the use of nutritional status indicators recommended by the WHO highlight the internal and external validity of the results on the temporal variation of obesity prevalence and the consumption of ultra-processed foods. The strategy used to assess the impact of temporal changes in the consumption of ultra-processed foods on obesity trends stands out as another advantage. Here, we used estimates obtained from regression models adjusted for multiple covariates and derived from the same databases that provided the time trend information, instead of using data from international studies published in the literature.

In conclusion, this study showed, in an unprecedented way, that the increase in the consumption of ultra-processed foods in the period of 2002–2009 was responsible for more than a fourth of the increase in the prevalence of obesity in the same period in Brazil. These results reinforce the importance of implementing actions aimed at improving food systems in the country.
