# Corrigendum: Changes in Obesity Prevalence Attributable to Ultra-Processed Food Consumption in Brazil Between 2002 and 2009

**DOI:** 10.3389/ijph.2022.1605178

**Published:** 2022-08-08

**Authors:** Maria Laura Louzada, Eurídice Martinez Steele, Leandro F. M. Rezende, Renata Bertazzi Levy, Carlos Augusto Monteiro

**Affiliations:** ^1^ School of Public Health, University of São Paulo, São Paulo, Brazil; ^2^ Center for Epidemiological Research in Nutrition and Health, School of Public Health, University of São Paulo, São Paulo, Brazil; ^3^ Departamento de Medicina Preventiva, Escola Paulista de Medicina, Universidade Federal de São Paulo, São Paulo, Brazil; ^4^ Faculty of Medicine, University of São Paulo, São Paulo, Brazil

**Keywords:** obesity, Brazil, ultra-processed foods, population attributable fraction, epidemiology, nutrition surveys

In the original article, there was a mistake in Table 3 as published. The 95% CI in the last column is “0.88 to 2.29” and not “0.33 to 1.59”. The corrected Table 3 appears below.

**TABLE 3:** Prevalence of obesity predicted from two multiple-adjusted linear regression models^a^ according to alternative scenarios regarding the consumption of ultra-processed foods. Brazilian households strata in 2002/3 (*n* = 443) and 2008/9 (*n* = 550).

Survey year	Population distribution of consumption of ultra-processed foods as in	Predicted multiple-adjusted prevalence of obesity (%)		Population distribution of consumption of ultra-processed foods as in	Predicted prevalence of obesity (%)		Difference between predicted values	
		Mean	95% CI		Mean	95% CI	Mean	95% CI
2002/3	2002/3	10.15	9.79; 10.53	2008/9	10.53	10.38; 10.69	0.38	−0.14 to 0.90
2008/9	2002/3	12.55	12.26; 12.84	2008/9	14.11	13.66; 14.55	1.55	0.88 to 2.29

a Models adjusted for household income per capita, setting (urban/rural), region of the country, percentage of expenditure on eating out of home, proportion of children, women and the elderly in stratum.

The authors apologize for this error and state that this does not change the scientific conclusions of the article in any way. The original article has been updated.

