# Supplementary figures and images for: Changes in Obesity Prevalence Attributable to Ultra-Processed Food Consumption in Brazil Between 2002 and 2009
Source: Int J Public Health. 2022 May 20;67:1604103. doi: 10.3389/ijph.2022.1604103 (PMC9163957; doi:10.3389/ijph.2022.1604103)

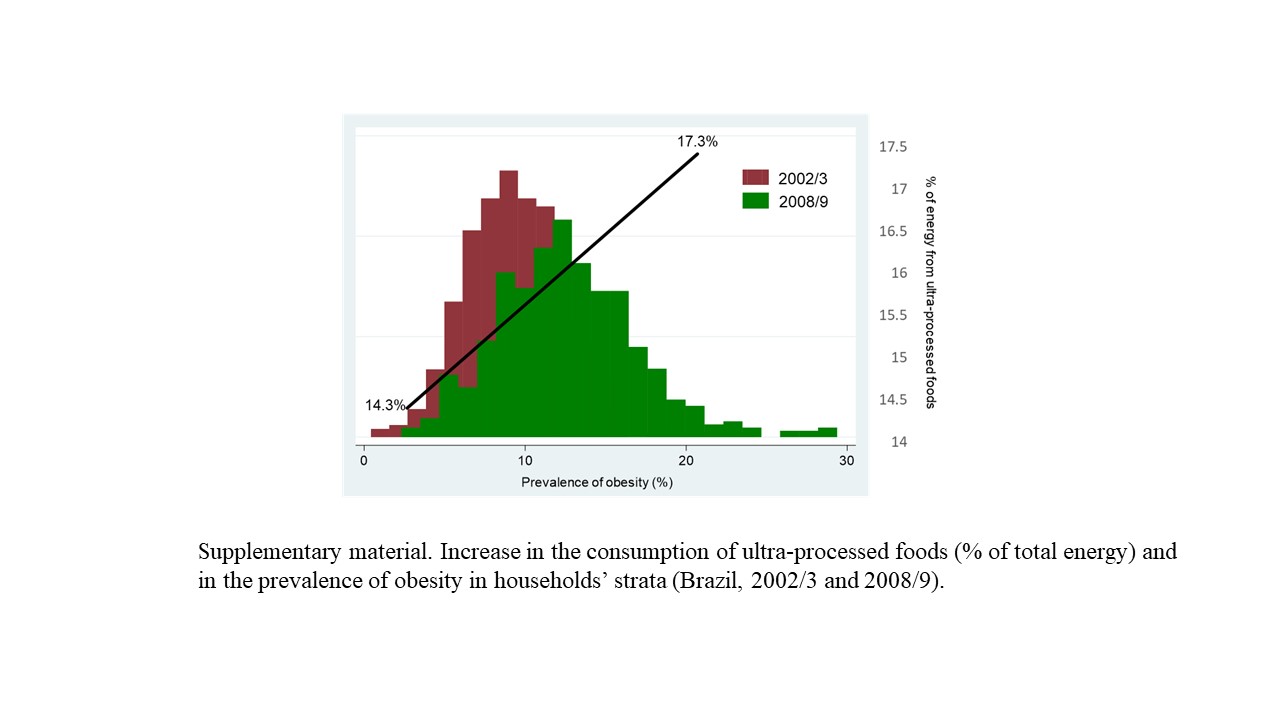

Supplement: Supplementary file 1 [file Image1.JPEG]
